# Consumption Simulations Induce Salivation to Food Cues

**DOI:** 10.1371/journal.pone.0165449

**Published:** 2016-11-07

**Authors:** Mike Keesman, Henk Aarts, Stefan Vermeent, Michael Häfner, Esther K. Papies

**Affiliations:** 1 Department of Psychology, Utrecht University, Utrecht, the Netherlands; 2 Communication Psychology, Berlin University of the Arts, Berlin, Germany; 3 Institute of Neuroscience and Psychology, University of Glasgow, Glasgow, United Kingdom; The University of Tokyo, JAPAN

## Abstract

Salivation to food cues is typically explained in terms of mere stimulus-response links. However, food cues seem to especially increase salivation when food is attractive, suggesting a more complex psychological process. Adopting a grounded cognition perspective, we suggest that perceiving a food triggers simulations of consuming it, especially when attractive. These simulations then induce salivation, which effectively prepares the body for eating the food. In two experiments, we systematically examined the role of simulations on salivation to food cues. As stimuli, both experiments used an attractive, a neutral, and a sour food, as well as a non-food control object. In Experiment 1, participants were instructed to simulate eating every object they would be exposed to. We then exposed them to each object separately. Salivation was assessed by having participants spit their saliva into a cup after one minute of exposure. In Experiment 2, we instructed half of participants to simulate eating each object, and half to merely look at them, while measuring salivation as in Experiment 1. Afterwards, participants rated their simulations and desire to eat for each object separately. As predicted, foods increased salivation compared to the non-food control object, especially when they were attractive or sour (Exp. 1 and 2). Importantly, attractive and sour foods especially increased salivation when instructed to simulate (Exp. 2). These findings suggest that consumption simulations play an important role in inducing salivary responses to food cues. We discuss directions for future research as well as the role of simulations for other appetitive processes.

## Background

Seeing a food such as chips can be enough to make your mouth water. Much research supports this link between food cues and increases in salivation (for reviews, see [1,2]). The explanations of how food cues lead to salivation typically relate to classical conditioning [2]: Much like Pavlov’s dog, people associate the smell of chips with the salivation produced in response to eating them. Later, the smell of chips produces a salivary response, even in the absence of actual consumption. However, salivation is stronger under certain circumstances compared to others. For instance, food cues induce more salivation when the food is liked more [3,4]. Unrestrained eaters salivate more to food cues than do restrained eaters (i.e. chronic dieters; [5]). Furthermore, hunger tends to increase salivation to food cues [6,7], although this relationship is not entirely clear [8]. Imagining a favorite food also increases salivation without any sensory exposure, especially when participants have vivid mental imagery (e.g. [4]). In summary, individuals seem to have especially strong salivary responses to food cues when the food is attractive. This suggests that the relation between food cues and salivation is more complex than a mere stimulus-response link.

**Fig 1:**
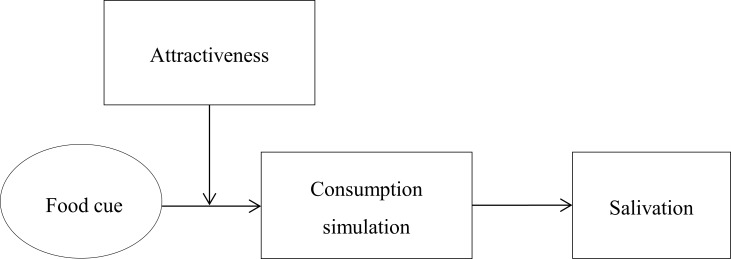
Overview of our grounded cognition account of salivation to food cues.

In the current paper, we examine whether consumption simulations induce salivary responses to food cues. Specifically, we suggest that food cues trigger reenactments of earlier eating experiences, especially when the food is attractive, which then induce salivation. We refer to such reenactments as *simulations* [9,10]. Fig 1 gives an overview of this account of salivary responses to food cues, which is derived from grounded cognition [11]. This grounded account of salivation might also function to more generally predict and explain the effects of rewarding stimuli on appetitive responses. The current research, however, was designed to examine this in the context of salivation to food cues.

### Salivation from the perspective of grounded cognition

Adopting a grounded cognition perspective, food cues trigger consumption simulations [12,13]. This process of eliciting simulations is largely in line with models of associative networks (e.g. [14]): Earlier eating experiences are stored as representations in memory, and when any property of such a representation gets activated, this may reactivate associated features through pattern completion inferences [15,16]. A representation that best fits the current situation gets activated [17]. As food experiences typically involve their consumption, food cues are therefore likely to elicit simulations of consumption. Seeing chips, for instance, may trigger simulations of their salty taste and the enjoyment of eating them [18]. These simulations usually occur automatically and unconsciously, but they can be facilitated when people are induced to actively engage in or focus attention on them [19]. A recent review of fMRI research on the neural processes underlying food perception and food consumption corroborates the notion that food cues trigger simulations of earlier eating experiences [20]. In these experiments, participants consumed a food in the fMRI scanner, or they were presented with a food word or picture. The results showed that the activated brain areas were similar for eating and perceiving a food. Both, for instance, activated taste and reward areas. Similar neural processes are thus active for both eating and perceiving food. This offers additional evidence that simply perceiving a food cue triggers simulations of earlier eating experiences.

Importantly, food cues are especially likely to trigger simulations of consumption when the food is considered attractive [12, 18]. A food may be considered attractive when the features related to consuming it are salient and rewarding, such as a pleasant taste or texture, or the positive affect of eating with friends. Similarly, when hungry, sensory and reward experiences of eating are amplified [21,22], which makes the food seem more attractive. These consumption experiences are stored in the representations of the food, and when subsequently cued with the food, they are *reenacted* as simulations. Both fMRI and behavioral research corroborate that attractive foods trigger more consumption-related simulations than neutral foods [20,23]. Furthermore, consumption simulations are modulated by situational factors and goals, such that they are increased when hungry and decreased when a person holds a dieting goal that precludes consumption [13,20]. Taken together, this evidence clearly supports the perspective that food cues especially induce simulations of consumption when the food is considered attractive.

When actually eating a food, the body produces a salivary response [24,25]. We suggest that simulations of eating a food produce salivary responses similar to when a food is actually being eaten. This produced saliva is useful for chewing, swallowing, and digesting food [25]. Saliva has an additional function for sour food, as it protects the digestive system by diluting harmful acids that may for instance cause dental erosion [25,26]. Accordingly, sourness of a food increases salivation [27]. Salivation thus facilitates the consumption of a food. Similarly, when simulations of consumption induce salivation, the body effectively prepares for and facilitates upcoming consumption of the food.

With this process of simulation, the grounded model of salivation to food cues can be used to explain the modulating effects of food properties, situational factors, and goals, on salivation that were described above. For example, we expect attractive compared to neutral foods to trigger increased simulations of consumption, which subsequently induce increased salivation. Furthermore, simulations are less likely to be triggered if an individual is trying to diet, when satiated, or when suffering from anorexia nervosa [12,20]. Our account predicts that these conditions therefore reduce salivary responses. Indeed, individuals suffering from anorexia nervosa salivate less to food cues than a control group [28]. Consumption simulations might similarly explain and predict other embodied reactions to food cues. One such reaction is the release of the hormone ghrelin, which increases appetite and prepares the body to eat by triggering gastric contractions that aid digestion [29–31]. Parallel to the expected increase in salivation for attractive compared to neutral food in the current research, ghrelin is increased when participants are cued with an indulgent compared to healthy milkshake [32]. This suggests that ghrelin is induced by a more complex psychological process than a direct stimulus-response link. We suggest the mechanism is similar to that of salivation: food cues trigger simulations of eating the food, which then induce the release of ghrelin. Simulations then generally work to prepare the body to eat, not just by inducing salivation, but also leading to other bodily responses reactions, such as by releasing ghrelin and inducing an approach impulse to pick up the food. Importantly, if this is the case, the grounded model with simulation as its central mechanism could be used to better predict appetitive responses to food cues more generally.

### Overview of the current research

In the current research, we systematically tested whether consumption simulations induce salivation, and thus whether the link between food cues and salivation is more complex than a mere direct stimulus-response link. We assessed salivation to an attractive food, a neutral food, a sour food, and a non-food control object. In previous experiments examining salivation in response to food cues, researchers often instructed participants to simulate eating the food [3,33–35]. Participants have been instructed, for instance, to “imagine as vividly as (you can) that (you are) actually eating the food”, “imagine its taste and its texture in your mouth”, and “give in to the feelings the pictures elicit.” As a proof of concept of our methodology, in our Experiment 1, we also instructed participants to simulate consuming any object that they would be exposed to. The salivary responses were tested in a within-participants design to increase statistical power, as salivary responses are highly stable within individuals [36,37]. We hypothesized that more saliva would be induced for the neutral food compared to the non-food control object, and more saliva for the attractive compared to neutral food. We further tested the hypothesis that sour food leads to the most salivation.

Experiment 2 took our test one step further by examining whether simulations of consumption induce salivation in response to food cues. The same objects were included as in Experiment 1. This time, half of participants were instructed to simulate consuming the presented objects, and the other half of participants were not. This allowed us to directly test whether simulating consumption increased salivation. Furthermore, we included a measure of consumption simulations and of desire to eat. As in Experiment 1, we hypothesized that more saliva would be induced in response to the foods than the non-food control object, especially when they were attractive or sour. Importantly, we hypothesized that salivation for the attractive and sour foods would be especially increased when participants simulated their consumption. For the attractive food, we furthermore expected to find a correlation between salivation and desire to eat, as both may indicate reactivity to food cues [34,38,39].

## Experiment 1

### Methods

#### Design and participants

We recruited 20 participants (5 males; age *M* = 20.96, *SD* = 2.26). We determined this sample size with an a priori power analysis with α at .05, and 95% power to detect the difference in salivation during food exposure compared to salivation at baseline as reported by Hardman and colleagues [33]. Participants could only sign up for participation if they did not smoke, if they liked chips (the selected attractive food), and if they agreed not to eat one hour before participation. Only non-smokers were recruited as smoking decreases salivary flow [40,41]. We used a within-participants design with four types of objects (attractive food, neutral food, sour food, non-food control object) and salivation as the dependent variable.

#### Materials

As stimulus materials, we used a small bag of chips (attractive food), a slice of bread with cheese (neutral food; typical Dutch breakfast food), a lemon slice (sour food), and a block of wood (non-food control object; [42]). Each participant received a new bag of chips, which was opened and put in a bowl in front of the participants. A fresh slice of lemon was cut in front of each participant, and was then put on a plate. During the experiment, participants did not eat any of the items.

#### Simulation instructions

Participants were instructed to simulate eating each of the objects as follows: “We want you to focus on the object as much as possible. Use the entire minute to focus on the following: Pick up the object. How does it feel? How does the object smell? How would the object taste? What would it be like to have this object in your mouth?” [33].

#### Saliva collection

For the collection of saliva, we used plastic cups that were marked and weighed using a 0.01-gram precision scale. Before collection started, we instructed participants to swallow. Then, we instructed them to keep their lips closed, to keep their tongue unmoved, and to refrain from swallowing for one minute. After one minute of stimulus exposure, participants collected their saliva and spit it out in the plastic cup, which was subsequently weighed by the experimenter. We instructed participants to not eat any of the items during the experiment, but they could eat it at a later time if they wished.

#### Procedure

Participants read and signed the informed consent form, and were asked to rinse their mouth with a cup of water. Then, they reported their current hunger and thirst. The experimenter told participants that they would be exposed to various objects and gave them the simulation instruction. Then, the saliva collection procedure was explained to them. To probe for understanding, participants were asked to repeat what was explained to them.

**Fig 2:**
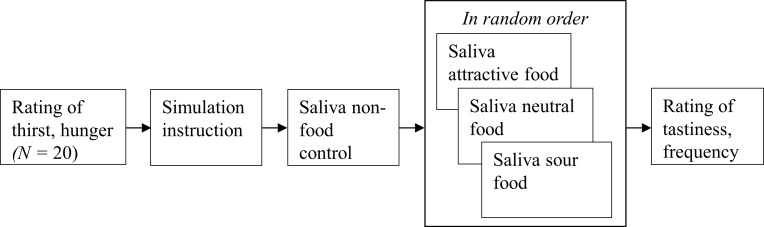
Overview of the procedure of Experiment 1.

Participants were first exposed to the non-food control object, and a saliva sample was taken. A 3 minute break followed during which participants could read chapter one of the popular novel “The Hobbit” [43]. This procedure was then repeated for the three foods, to which each participant was exposed to in random order. After the saliva collection for all items, participants rated each food on tastiness and frequency of consumption. This was done on scales from 1 (not at all tasty/never, respectively) to 7 (very tasty/very often, respectively). See Fig 2 for an overview of the procedure.

#### Ethics statements

All participants provided written informed consent before participating in this study. In the Netherlands, IRB approval must be obtained for research involving human participants when it concerns medical-scientific research, contains infringement of participants’ physical or psychological integrity, or involves deception. This was not the case in the current research, and therefore no IRB approval was requested, see S1 Waiver of Approval.

### Results

#### Descriptive statistics

Participants had a healthy BMI of 21.47 (*SD* = 2.49). Participants rated their hunger to be around the midpoint of the scale, at 4.05 (*SD* = 1.32).

#### Outlier removal

We considered data points that differed by more than 3 standard deviations from the mean as outliers. In this Experiment, there were no outliers.

#### Manipulation check

The attractive food (*M* = 5.80, *SD* = .83) was considered tastier than the neutral food (*M* = 3.90, *SD* = 1.68; *t*(19) = 4.20, *p* < .001) and the sour food (*M* = 4.25, *SD* = 1.68; *t*(19) = 3.28, *p* = .004). This indicates that our attractiveness manipulation was successful.

#### Main analyses

**Fig 3:**
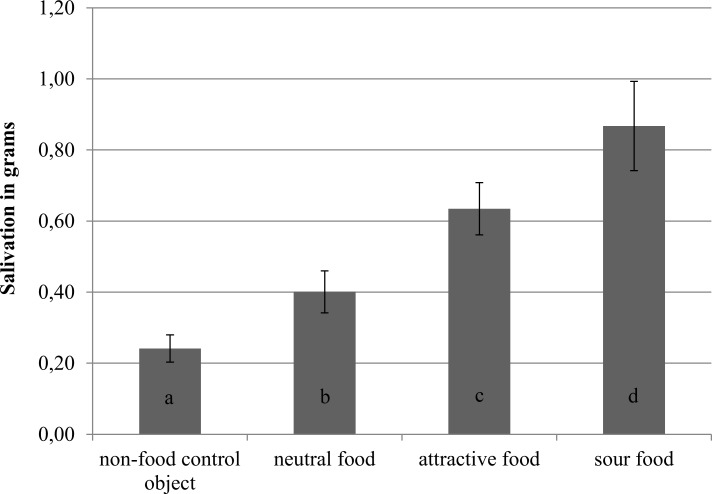
Salivation in response to stimulus exposure and simulating its consumption in Experiment 1. Different letters indicate significant differences between objects, *p* < .05. Error bars represent standard errors from the mean.

In line with our hypotheses, foods induced more salivation than the non-food control object, especially when attractive or sour, as can be seen in Fig 3. Paired t-tests indicated that there was significantly more salivation for the neutral food than for the non-food control object, *t(*19) = 3.73, *p* = .001, *d* = .83 (with 95% CI .31,1.33). Furthermore, more saliva was produced for the attractive than for the neutral food, *t*(19) = 5.74, *p* < .001, *d* = 1.29 (with 95% CI .68,1.87). More saliva was also induced for the sour compared to attractive food, *t*(19) = 2.51, *p* = .021, *d* = .56 (with 95% CI .08, 1.02). In sum, foods triggered salivary responses, especially when the food was attractive or sour, and the sizes of these effects were large.

There were no main or interaction effects of order, all *F* < 1.62, *p* > .16.

#### Further analyses

There were no main or interaction effects of gender or of self-reports of hunger on salivation to the foods (neutral, attractive, sour), all *F* < 1.

### Discussion

The results confirmed our hypotheses that people salivate more to foods than to a non-food control object, especially when they are attractive or sour. The finding that more saliva was induced for the sour compared to neutral food replicates other previous experiments [27,39]. Although the effect of food attractiveness on increased salivation has not been examined often, our findings do replicate this limited earlier work [3,4]. The effect of food attractiveness on increased salivation cannot be explained by mere stimulus-response links, but fits our grounded model, with simulation as the process that induces salivation.

## Experiment 2

Experiment 2 was designed to examine whether consumption simulations induce salivation to food cues. The procedure was largely similar as for Experiment 1, but now half of participants were instructed to simulate eating each item, and half were instructed to merely look at them. This allowed us to directly test the effect of simulating consumption on salivation to food cues. Additionally, after measuring salivation as in Experiment 1, participants rated their simulations and desire to eat for each object separately. We again hypothesized that food cues would increase salivation, especially when the food was attractive or sour. Importantly, we hypothesized the instruction to simulate to increase the effect of object type on salivation, relative to the mere exposure instruction. We further expected salivation and desire to eat to be correlated for the attractive food.

### Methods

#### Design and participants

We recruited 60 participants (17 male; mean age = 21.17, *SD* 2.29). An a priori power analysis with α at .05 and 95% power indicated that a sample size of 11 would be required to detect the difference in salivation between the attractive and neutral food from Experiment 1. As the effect sizes for the mere exposure condition were unknown, we chose to collect data from 30 participants per group, and thus from 60 in total. This allowed us to detect an effect size of *d* = .68, about half the effect size we found in Experiment 1 for the difference in salivation to the attractive compared to neutral food. Again, participants could only sign up for participation if they had not participated in Experiment 1, did not smoke, liked chips (the selected attractive food), and if they agreed not to eat one hour before participation. The experiment had a 2 (condition: instructed simulation, mere exposure; between participants, with random assignment) x 4 (object type: attractive food, neutral food, sour food, non-food control object; within participants) mixed design with salivation as the dependent variable.

#### Conditions

Participants in the instructed simulation condition received the instruction to simulate eating each of the presented objects as in Experiment 1. Participants in the mere exposure condition received the instruction to “focus on the object for one minute. We will ask you some questions about it later”. Thus, no mention was made of simulation, consumption, or touching any of the items.

#### Measure of simulations

For each item, participants reported the extent to which they had experienced simulations related to food consumption. This was done using 5 statements to which participants responded on a scale from 1 (not at all) to 10 (definitely; “I imagined that I was eating the object”, “It was as if I could really taste the object”, “It was as if I could really feel the texture of the object in my mouth”, “I imagined how it would be to eat the object”, “I imagined how eating this object would make me feel”; based on [44]). A measure of simulations was created using the average score of the 5 items, *α* > .84 for each of the foods.

#### Desire to eat

For each item, participants were asked to respond to the statement “I would have liked to eat (the item)” on a scale from 0 (not at all) to 10 (very much).

#### Concern for dieting

To assess concern for dieting, a 6-item subscale of the restraint scale with statements such as “I diet….” was administered [45]. The response options ranged from 0 (never) to 3 (always) and 0 (not at all) to 4 (strongly), *α* = .76.

#### Procedure

**Fig 4:**
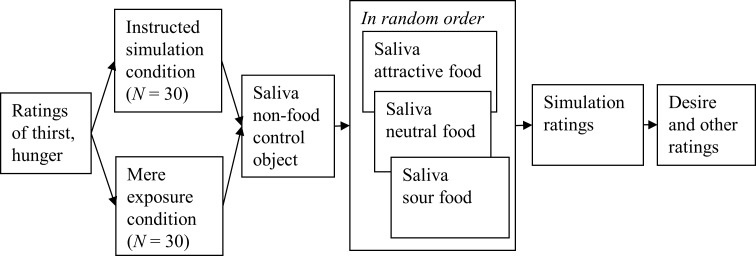
Overview of the procedure of Experiment 2.

The experiment largely followed the procedure of Experiment 1; see Fig 4 for an overview. First, ratings of participants’ current hunger and thirst were obtained as in Experiment 1. The experimenter then told participants that they would be exposed to various objects. Then, participants received the simulation or mere exposure instruction. Salivation was assessed as in Experiment 1, first for the non-food control object, and then in random order for each of the three foods. After saliva collection was finished, simulation ratings were obtained for each object, and then ratings of desire to eat. As in Experiment 1, participants rated the tastiness of each food, but also rated the foods on other properties such as sourness and crunchiness. This was done on scales from 1 (not at all) to 7 (very much). Then, participants completed the concern for dieting scale. Finally, all participants were thanked for their participation and received remuneration.

#### Ethics statements

All participants provided written informed consent before participating in this study. In the Netherlands, IRB approval must be obtained for research involving human participants when it concerns medical-scientific research, contains infringement of participants’ physical or psychological integrity, or involves deception. This was not the case in the current research, and therefore no IRB approval was requested, see S1 Waiver of Approval.

### Results

#### Descriptive statistics

Table 1 displays the descriptive statistics, showing that participants had a healthy BMI, low concern for dieting, and medium levels of hunger.

**Table 1:** Descriptive statistics of the participants in Experiment 2, by condition.

	Instructed simulation (*N* = 30)	Mere exposure (*N* = 30)	Differences
BMI	*M* = 21.40 (*SD* = 2.20)	*M* = 21.27 (*SD* = 1.92)	*t* < 1, *p* = .80
Concern for dieting (range: 0–20)	*M* = 6.63 (*SD* = 3.08)	*M* = 5.90 (*SD* = 3.40)	*t <* 1, *p =* .38
Hunger (range: 1–7)	*M* = 4.03 (*SD* = 1.35)	*M* = 4.03 (*SD* = 1.38)	*t* < 1, *p* = .99

#### Outlier removal

We considered data points that differed by more than 3 standard deviations from the mean as outliers, and did not include them in our analyses. Outlier removal did not influence our main results. Two participants had outliers for the measures of salivation and simulation, for one participant there was an outlier for desire to eat.

#### Manipulation check

The attractive food (*M* = 5.78, *SD* = 1.62) was considered tastier than the neutral food (*M* = 4.28, *SD* = 1.34; *t*(59) = 6.12, *p* < .001) and the sour food (*M* = 3.82, *SD* = 1.63; *t*(59) = 6.67, *p* < .001). Furthermore, the sour food (*M* = 6.22, *SD* = .94) was considered more sour than the neutral food (*M* = 1.30, *SD* = .74; *t*(59) = 34.33, *p* < .001) and the attractive food (*M* = 1.23, *SD* = .72; *t*(59) = 34.71, *p* < .001). These analyses suggest that our attractiveness and sourness manipulations were successful.

**Fig 5:**
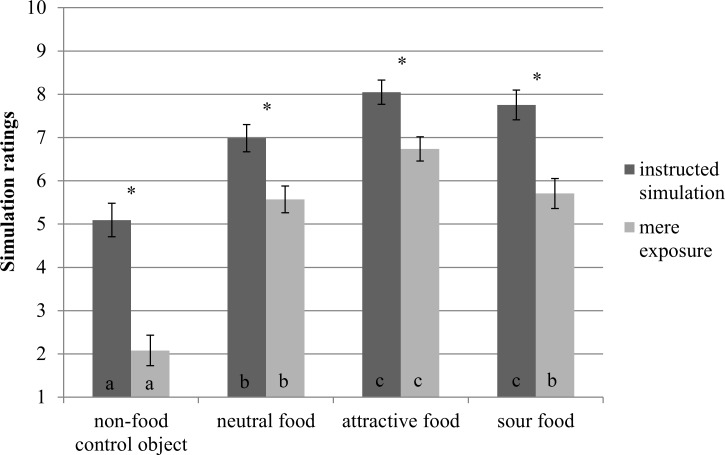
Ratings of consumption simulations by object type and condition in Experiment 2. Different letters indicate significant differences between objects within a condition, *p* < .05. Asterisks indicate significant differences between conditions for an object, *p* < .05. Error bars represent standard errors from the mean.

Importantly, participants in the instructed simulation condition reported more consumption simulations than those in the mere exposure condition, *F*(1,56) = 31.93, *p* < .001, ηp^2^ = .36. Furthermore, in the absence of explicit simulation instructions, participants reported increased simulation ratings for the attractive food compared to the other foods, all within participant contrasts, *F*(1,27) > 12.05, *p* < .003, ηp^2^ > .30. These results indicate that our manipulation of instructed simulation was successful. Additionally, the attractive food triggered the most consumption simulations. See Fig 5 for an overview of the simulation ratings.

#### Main analyses

**Fig 6:**
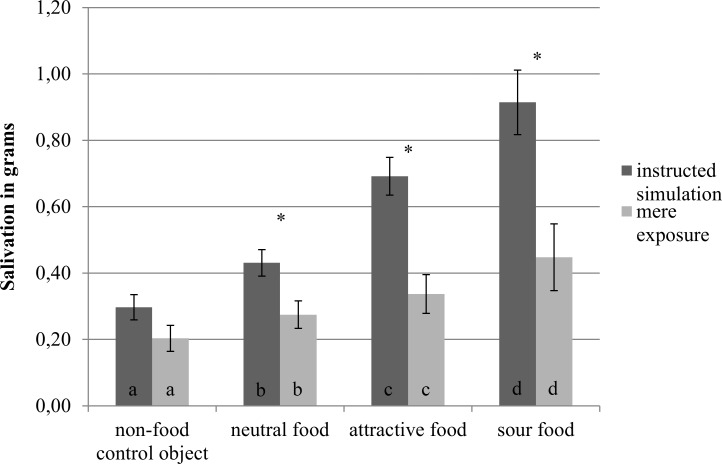
Salivation by object type and condition in Experiment 2. Different letters indicate significant differences between objects within a condition, *p* < .05. Asterisks indicate significant differences between conditions for an object, *p* < .05. Error bars represent standard errors from the mean.

We replicated our findings from Experiment 1 that foods induce more salivation than a non-food control object, especially when attractive or sour. Participants in the instructed simulation condition salivated significantly more to the neutral food than to the non-food control object, *t*(29) = 4.69, *p* < .001, *d* = .86 (with 95% CI .43, 1.27). More saliva was also produced for the attractive than for the neutral food, *t*(29) = 6.18, *p* < .001, *d* = 1.13 (with 95% CI .66, 1.58), and more for the sour than attractive food, *t*(29) = 2.25, *p* = .032, *d* = .41 (with 95% CI .03, 78). The same pattern of results was found in the mere exposure condition. Participants salivated more to the neutral food than the non-food control object, *t*(27) = 4.29, *p* < .001, *d* = .81 (with 95% CI .38, 1.23). More saliva was also produced for the attractive than for the neutral food, *t*(28) = 3.04, *p* = .005, *d* = .53 (with 95% CI .13, .92), and more for the sour than attractive food *t*(28) = 2.90, *p* = .007, *d* = .58 (with 95% CI .17, 98). In sum, foods triggered salivary responses, especially when the food was attractive or sour. An overview of these findings can be seen in Fig 6.

Importantly, for attractive and for sour food compared to neutral food, salivation was especially increased when participants were instructed to simulate consumption. This was confirmed by the predicted interaction of condition with object type, *F*(3,168) = 8.27, *p* < .001, ηp^2^ = .13. With unequal variance t-tests, we found the instruction to simulate consumption to increase salivation for the attractive food, *t*(46.32) = 3.97, *p* < .001, *d* = 1.03 with 95% CI [.48, 1.58], the neutral food, *t*(52.06) = 2.43, *p* = .019, *d* = .63 (with 95% CI .10, 1.15), and the sour food, *t*(44.08) = 2.95, *p* = .005, *d* = .76 (with 95% CI .23, 1.29), but not for the non-food control object, *t*(57) = 1.27, *p* = .21, *d* = .33 (with 95% CI -.19, .84). Thus, the instruction to simulate increased salivation, and it modulated the effect of object type on salivary responses such that attractive and sour food especially increased salivation compared to neutral food when simulating its consumption.

As in Experiment 1, there were no main or interaction effects of order on salivation, all *F* < 1.

Finally, salivation and the desire to eat were correlated for the attractive food, *r*(54) = .35, *p* = .008, but not for the neutral and sour food, respectively *r*(54) = .16, *p* = .23, and *r*(54) = .16, *p* = .24. When computing these correlations, we controlled for salivation for the non-food control object.

#### Further analyses

**Fig 7:**
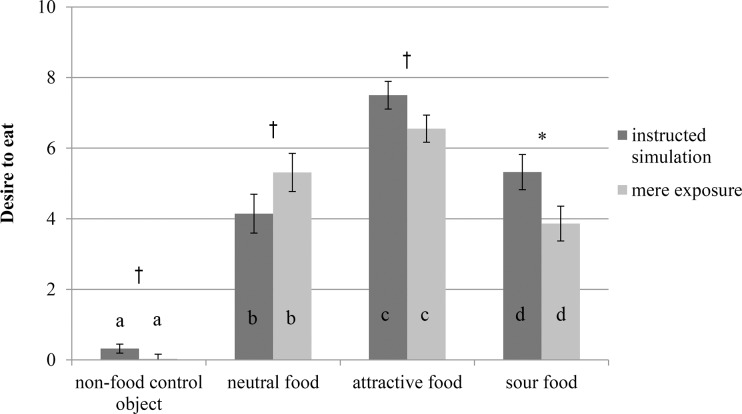
Desire to eat by object type and condition in Experiment 2. Different letters indicate significant differences between objects within a condition, *p* < .05. Asterisks indicate significant differences between conditions for an object, *p* < .05, and daggers indicate that *p* < .10. Error bars represent standard errors from the mean.

Participants experienced more desire to eat for the attractive food than for the neutral food, sour food, and non-food control object, all these contrasts, *F*(1,55) > 34.88, *p* < .001, ηp^2^ > .38. There was no main effect of condition on the variable assessing desire to eat, *F* = 1.28, *p* = .26, but there was an interaction effect of condition with object type, *F*(3,165) = 4.02, *p* = .009, ηp^2^ = .07. With unequal variance t-tests, we found the instruction to simulate consumption to somewhat increase the desire to eat the attractive food, *t*(49.70) = 1.79, *p* = .080, *d* = .45 (with 95% CI -.06, .97). The instruction to simulate further increased the desire to eat the sour food *t*(58) = 2.09, *p* = .041, *d* = .54 with 95% CI (.02, 1.05), and somewhat increased the reported desire to eat the non-food control object, *t*(30.01) = 1.86, *p* = .072, *d* = .44 (with 95% CI -.04, 1.01). However, it somewhat decreased the desire to eat the neutral food, *t*(52.85) = -1.78, *p* = .080, *d* = -.46 (with 95% CI -.97, .06). An overview of these findings can be found in Fig 7.

There was no main effect of hunger on salivation, nor did hunger interact with condition or object type, all *F* < 1.

There was also no main effect of concern for dieting on salivation, nor did concern for dieting interact with condition or object type, all *F* < 1.

There was a main effect of gender on overall salivation, such that men salivated significantly more (*M* = .57, *SE* = .08) than women (*M =* .40, *SE* = .05), *F*(1,37) = 4.52, *p* = .040, ηp^2^ = .11. However, gender did not interact with object type or condition, or their interaction, all *F* < 1.3, *p* > .27.

### Discussion

Replicating Experiment 1, the findings of Experiment 2 showed that foods led to stronger salivary responses than a non-food control object. Salivary responses were further increased for attractive and sour food. Importantly, these effects of object type were stronger when participants were instructed to simulate consumption. Instructed simulation further increased salivation in response to the foods, but not to the non-food control object. Salivation to the attractive food was also associated with the desire to eat it. The main findings confirm our account that consumption simulations play a crucial role in inducing salivary responses to food cues.

The instruction to simulate seemed to increase the desire to eat the attractive and sour food and to decrease the desire to eat the neutral food, but no strong conclusions can be drawn from these results. More conclusive evidence regarding the effect of a simulation manipulation on desire could be obtained in an experiment with increased statistical power, and by assessing desire directly after stimulus exposure instead of at the end of the experiment. We found no effect of concern for dieting on salivation, which is in line with some previous studies [3], but differs from others [5,46]. The absence of a moderating effect of concern for dieting may be due to participants’ generally low concern for dieting.

## General Discussion

In the current research, we found evidence for a grounded account of salivation to food cues, suggesting a crucial role of consumption simulations for inducing salivation. In both Experiment 1 and 2, foods increased salivation compared to a non-food control object, especially when the food was attractive or sour. Importantly, in Experiment 2, attractive and sour foods especially increased salivation when participants were instructed to simulate consumption. Overall, the instruction to simulate increased salivation to the foods compared to the mere exposure instruction. Salivation to the attractive food was additionally associated with the desire to eat it. The results thus show that simulations of consumption increase salivary responses to food cues.

Simulation is a basic psychological process [15], and we expect simulations of consumption to trigger appetitive responses to rewarding stimuli in various domains, such as alcohol consumption, sex, and smoking [12]. In line with this, a recent meta-analysis suggests that similar brain regions are active when cued with food and cigarettes, compared to neutral stimuli [47]. This suggests that food and cigarette cues induce similar psychological processes, such that both induce simulations of consumption. Furthermore, an fMRI study found that alcohol cues induce reward simulations, and that these were associated with craving in individuals that are addicted to alcohol [48]. More research is needed, however, to confirm whether consumption simulations induce such appetitive responses. A comprehensive framework of how appetitive responses are induced, such as the presented grounded framework, contributes to better understanding and predicting when appetitive responses arise.

If simulations of consumption work to trigger appetitive responses more generally, interventions to reduce undesired appetitive responses could be made more effective by targeting these simulations. This is because interventions are most effective when they prevent appetitive motivation rather than when they help resisting a current appetitive motivation [49]. For instance, not eating chips or not smoking a cigarette is easier when no appetitive motivation arises in the first place than when this motivation needs to be controlled and resisted. An experience sampling study on temptations portrays the magnitude of this issue [50]. Participants in this study were prompted 7 times per day, for 7 consecutive days, to respond to questions of whether they currently experienced temptations. On 76.6% of these prompts, participants indicated to currently experience or to have recently experienced an appetitive motivation, such as for food, for cigarettes, or to use social media. Importantly, participants enacted on 48% of these temptations, which could be predicted from the strength of their reported appetitive motivation. As we suggest that simulations of consumption induce appetitive responses, modulating these simulations is an important starting point for interventions. The priming of health goals is one approach to reduce simulations of consumption [20], which then subsequently prevents the appetitive behavior [51,52]. Overall, the grounded framework may be a useful starting point for the development of interventions to prevent undesired appetitive responses (see also [51]).

As a final note, it is important to stress that in psychological research, salivation to food is often taken as a proxy for desire to eat, where both indicate reactivity to food cues [34,38,39]. However, research does not consistently find an association between salivation and this desire to eat the food [5,53,54]. Our finding suggests that there may indeed be an association between salivation and desire to eat. Such an association might be explained from the perspective of grounded cognition, which suggests consumption simulations induce desire to eat [12], as well as salivation to food cues. Furthermore, to the extent that people are consciously aware of their salivary responses to food cues, they may infer that they want to eat it, which may increase their subjective experience of desire [55]. Accordingly, the association between desire to eat and salivation fits with grounded cognition and psychological construction. More research is needed to confirm this association, for instance using behavioral or implicit measures of desire rather using self-reports at the end of the experiment.

A limitation of the current research is that we could not disentangle the visual and olfactory sources that contributed to the difference in salivation between each of the foods. Furthermore, we only obtained one saliva sample for each type of food, which precludes us from generalizing to other foods. This is common in research on salivation to food cues, with many studies having only assessed salivation at baseline (without any objects) and to one food item. One reason for researchers to obtain only a limited number of saliva samples might be that salivation might be decreased when obtaining many such samples. This might be especially likely when using the standard method of obtaining saliva samples of using up to three cotton rolls to absorb the saliva in a participant’s mouth. These cotton rolls might leave a dry mouth, and participants might also find it a somewhat aversive experience, which could then lead to decreased salivation on subsequent trials. Using the spitting method, however, we showed that it is possible to obtain at least four saliva samples without order effects. Furthermore, we included a non-food object as a control stimulus, similar to Naumann and colleagues [42], which is important, but not yet a standard procedure in research on salivation to food cues. Future research could therefore benefit from the paradigm designed here with a control stimulus and the spitting method to obtain saliva samples.

To conclude, the present study examined motivated consummatory responses to food cues, and demonstrated that salivary responses are increased when people simulate the consumption of food that benefits from such responses. The role of simulations in consummatory behavior of food has been largely neglected so far as a potential source for under- and overregulation of eating behavior. We therefore hope that the present analysis might offer an interesting and fruitful avenue for further research in this important health domain.

## Supporting Information

S1 Waiver of Approval (PDF)
