# Supplementary material for: Consumption Simulations Induce Salivation to Food Cues
Source: PLoS One. 2016 Nov 7;11(11):e0165449. doi: 10.1371/journal.pone.0165449 (PMC5098730; doi:10.1371/journal.pone.0165449)
Supplement: S1 Waiver of Approval — (PDF) [file pone.0165449.s001.pdf]

Universiteit Utrecht

P.O. Box 80140, 3508 TC Utrecht

The Board of the Faculty of Social and Behavioural Sciences
Utrecht University
P.O. Box 80.140
3508 TC Utrecht

Faculty of Social and Behavioural Sciences
Faculty Support Office
Ethics Committee

Visiting Address
Padualaan 14
3584 CH Utrecht

**Our Description**	FETC16-067 (Keesman)
**Telephone**	030 253 46 33
**E-mail**	FETC-fsw@uu.nl
**Date**	July 7, 2016
**Subject**	Ethical approval

ETHICAL APPROVAL

Study: 'Twee experimenten naar de fundamentele psychologische processen onderliggend aan speekselproductie'

Principal investigators: Mike Keesman, MSc., prof. Henk Aarts, Ph.D., Esther Papies, Ph.D.

This research project does not belong to the regimen of the Dutch Act on Medical Research Involving Human Subjects, and therefore there is no need for approval of a Medical Ethics Committee.

The study is approved by the Ethics Committee of the Faculty of Social and Behavioural Sciences of Utrecht University. The approval is based on the documents send by the researchers as requested in the form of the Ethics committee and filed under number FETC16-067 (Keesman). Given the review reference of the Ethics Committee, there are no objections to execution of the proposed research project, as described in the protocol. It should be noticed that any changes in the research design oblige a renewed review by the Ethics Committee.

Yours sincerely,

Peter van der Heijden, Ph.D.
Chair

Jacqueline Tenkink-de Jong LLM
Executive secretary
